# Development of a Nanobody-Based Lateral Flow Immunoassay for Detection of Human Norovirus

**DOI:** 10.1128/mSphere.00219-16

**Published:** 2016-10-12

**Authors:** Sylvie Y. Doerflinger, Julia Tabatabai, Paul Schnitzler, Carlo Farah, Steffen Rameil, Peter Sander, Anna Koromyslova, Grant S. Hansman

**Affiliations:** a Schaller Research Group at the University of Heidelberg and the DKFZ, Heidelberg, Germany; b Department of Infectious Diseases, Virology, University of Heidelberg, Heidelberg, Germany; c Child and Adolescent Medicine, General Pediatrics, University Hospital Heidelberg, Heidelberg, Germany; d R-Biopharm AG, Darmstadt, Germany; University of Pittsburgh School of Medicine

**Keywords:** Nanobody, lateral flow assay, norovirus

## Abstract

Human noroviruses are the dominant cause of outbreaks of acute gastroenteritis. These viruses are usually detected by molecular methods, including reverse transcriptase PCR (RT-PCR) and enzyme-linked immunosorbent assay (ELISA). Human noroviruses are genetically and antigenically diverse, with two main genogroups that are further subdivided into over 40 different genotypes. During the past decade, genogroup 2 genotype 4 (GII.4) has dominated in most countries, but recently, viruses belonging to GII.17 have increased in prevalence in a number of countries. A number of commercially available ELISAs and lateral flow immunoassays were found to have lower sensitivities to the GII.17 viruses, indicating that the antibodies used in these methods may not have a high level of cross-reactivity. In this study, we developed a rapid Nanobody-based lateral flow immunoassay (Nano-immunochromatography [Nano-IC]) for the detection of human norovirus in clinical specimens. The Nano-IC assay detected virions from two GII.4 norovirus clusters, which included the current dominant strain and a novel variant strain. The Nano-IC method had a sensitivity of 80% and specificity of 86% for outbreak specimens. Norovirus virus-like particles (VLPs) representing four genotypes (GII.4, GII.10, GII.12, and GII.17) could be detected by this method, demonstrating the potential in clinical screening. However, further modifications to the Nano-IC method are needed in order to improve this sensitivity, which may be achieved by the addition of other broadly reactive Nanobodies to the system.

**IMPORTANCE** We previously identified a Nanobody (termed Nano-85) that bound to a highly conserved region on the norovirus capsid. In this study, the Nanobody was biotinylated and gold conjugated for a lateral flow immunoassay (termed Nano-IC). We showed that the Nano-IC assay was capable of detecting at least four antigenically distinct GII genotypes, including the newly emerging GII.17. In the clinical setting, the Nano-IC assay had sensitivities equivalent to other commercially available lateral flow systems. The Nano-IC method was capable of producing results in ~5 min, which makes this method useful in settings that require rapid diagnosis, such as cruise ship outbreaks and elder care facilities. The Nano-IC assay has several advantages over antibody-based IC methods: for example, Nanobodies can be readily produced in large quantities, they are generally more stable than conventional antibodies, and the Nanobody binding sites can be easily obtained by X-ray crystallography.

## Abstract (precis)

We previously identified a Nanobody (termed Nano-85) that bound to a highly conserved region on the norovirus capsid. In this study, the Nanobody was biotinylated and gold conjugated for a lateral flow immunoassay (termed Nano-IC). We showed that the Nano-IC assay was capable of detecting at least four antigenically distinct GII genotypes, including the newly emerging GII.17. In the clinical setting, the Nano-IC assay had sensitivities equivalent to other commercially available lateral flow systems. The Nano-IC method was capable of producing results in ~5 min, which makes this method useful in settings that require rapid diagnosis, such as cruise ship outbreaks and elder care facilities. The Nano-IC assay has several advantages over antibody-based IC methods: for example, Nanobodies can be readily produced in large quantities, they are generally more stable than conventional antibodies, and the Nanobody binding sites can be easily obtained by X-ray crystallography.

## OBSERVATION

Human noroviruses can cause both sporadic infections and outbreaks, often leading to epidemics and pandemics. Asymptomatic infections are not uncommon, and these individuals can be sources for further spread of norovirus (1). Rapid detection methods that can identify index cases could be important for reducing transmission, since there are few options that can promptly decontaminate an outbreak site, especially in large settings such as schools, hospital wards, and cruise ships.

Based on the capsid gene sequences, at least seven different norovirus genogroups (GI to GVII) have been assigned (2). The genogroups are further subdivided into numerous genotypes, and an association between genetic clusters and antigenicity is clear (3). In the past decade, a single genetic cluster (genogroup GII genotype 4 [GII.4]) has dominated (4). However, recently, a GII.17 variant norovirus was found to cause a large number of outbreaks in 2014 and 2015, and epidemiologists have indicated that the GII.17 noroviruses might replace the GII.4 norovirus (5).

The gold standard of detecting a norovirus infection is with reverse transcriptase PCR (RT-PCR) and sequence analysis (as well as real-time RT-PCR), yet these methods can take more than 3 h to perform. Other detection methods include enzyme-linked immunosorbent assay (ELISA) and lateral flow immunoassay (immunochromatography [IC]). The ELISA method is practical for screening a large number of specimens within 2 to 3 h, whereas the IC method can deliver results in ~5 min.

The commercial ELISA and IC methods are comprised of conventional antibodies (polyclonal and monoclonal) that are mainly developed against norovirus virus-like particles (VLPs). For the ELISA method, a sandwich format is mostly used and requires at least two antibodies (capture and detector), which need to be broadly reactive. For IC, only one broadly reactive antibody is needed, although several antibodies can be utilized in the assay. The main problem associated with antibody-based methods is that noroviruses are constantly evolving, and antibodies may not cross-react against new antigenic variants. For example, the GII.17 viruses were found to be less reactive in several commercially available IC methods (6).

In this study, we examined norovirus positive and negative stool specimens using RT-PCR, ELISA, and a novel IC method based on Nanobodies (termed Nano-IC). Stool specimens were collected at Heidelberg University Hospital, Germany. This included 32 specimens from an outbreak at Heidelberg University Hospital between 20 December 2013 and 11 February 2014 and 82 sporadic infections in Heidelberg between 12 April 2015 and 21 April 2015. All specimens were collected in the winter period and 1 to 4 days after the onset of symptoms.

Rotavirus, adenovirus, and astrovirus were screened using a commercial ELISA (Ridascreen). Norovirus specimens were screened using RT-PCR/sequencing, a commercially available ELISA (Ridascreen), a Nanobody-based ELISA (Nano-ELISA), and a novel Nanobody-based IC assay (Nano-IC). For RT-PCR, RNA extraction and RT-PCR were performed as previously described (7, 8). For norovirus GI, we used sense COG1F and antisense G1SKR primers. For norovirus GII, we used sense G2F3 and antisense G2SKR primers. Complete capsid nucleotide sequencing and phylogenetic analysis were performed as previously described (7).

**FIG 1:**
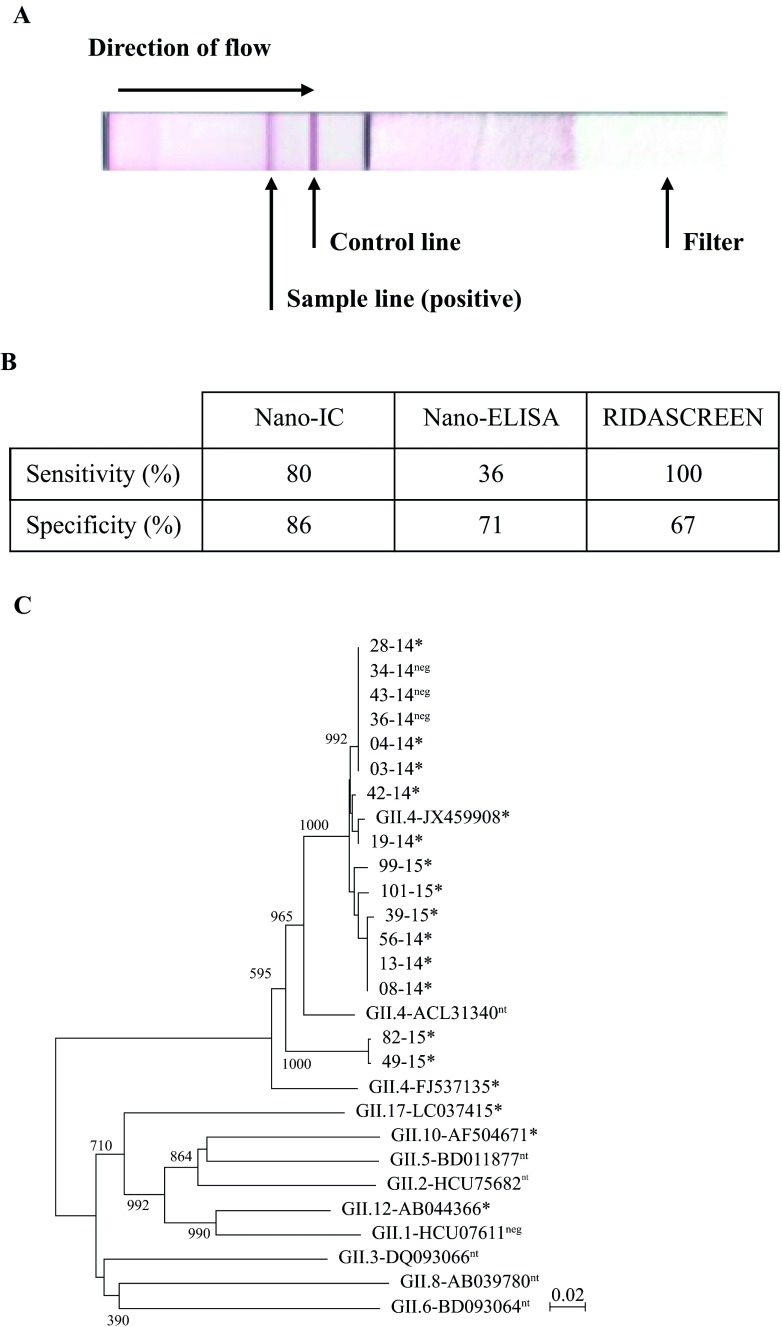
Analysis of Nano-85 binding to clinical norovirus specimens and VLPs. (A) Representative lateral flow strip assay showing the positive signal for norovirus detection. (B) Sensitivities and specificities of the Nano-IC, Nano-ELISA, and Ridascreen methods. (C) Phylogenetic analysis of the 17 isolated capsid sequences (amino acid) from the outbreak (numbered XX-14) and sporadic infections (numbered XX-15). The Nano-IC results are represented as follows: *, positive reading; neg, negative reading; and nt, not tested. Representative genotypes are also included in the phylogenetic analysis. The bootstrap values are shown on the branches.

The commercial ELISA was performed according to the manufacturer’s instructions (R-Biopharm AG). For the Nano-ELISA method, GII.4/GII.10 monoclonal antibodies were used as capture antigens and a Nanobody as the detector as previously described (9). The Nano-IC method comprises a typical lateral flow nitrocellulose membrane. The same Nanobody (Nano-85) used in the Nano-ELISA was incorporated into the Nano-IC assay and was biotinylated (0.02 µg/ml) and gold conjugated (0.02 µg/ml). The mobile solvent consists of 100 mM NaCl, 0.5% (wt/wt) bovine serum albumin (BSA), 0.25% (wt/wt) Triton X-100, and 40 mM Tris buffer (pH 7.5). The biotinylated Nano-85 and gold-conjugated Nano-85 sandwich the virus particle, and then the complex is immobilized at a streptavidin test line. A positive result is shown as a red line caused by the accumulation of the immune complexes, which consist of biotinylated Nano-85, norovirus particle, or gold-conjugated Nano-85 (Fig. 1A). The control line of the IC only appears when the assay was properly used. The stool specimens are first diluted (1:10) in phosphate-buffered saline (PBS) and then diluted (1:100) in the mobile solvent containing the labeled Nanobodies.

In the outbreak, one rotavirus, one adenovirus, and one astrovirus were detected. Norovirus was detected in 11 of 32 (34%) outbreak specimens using RT-PCR. The Ridascreen had a positive signal for 18 outbreak specimens. For the sporadic specimens, nine rotaviruses, five adenoviruses, and one astrovirus were detected. Norovirus was detected in 5 of 82 (7%) sporadic specimens using RT-PCR. The Ridascreen had a positive signal for eight sporadic specimens. Human sapovirus was not detected in any specimens using a previous RT-PCR method (10).

Based on RT-PCR results and sequence analysis (see below), the Nano-IC assay showed a true positive reading for 8 of 11 outbreak specimens and 3 of 5 sporadic specimens. The Nano-ELISA gave a true-positive signal for 4 of 11 outbreak specimens and 3 of 5 sporadic specimens. The sensitivities and specificities of the Nano-IC, Nano-ELISA, and Ridascreen methods for the outbreak are shown in Fig. 1B. The Nano-IC assay was superior over the Nano-ELISA, having sensitivities of 80% and 36%, respectively, while the Ridascreen showed 100% sensitivity. There were too few positive sporadic specimens to accurately determine the sensitivities and specificities of this collection. The viral load of the 17 positive specimens was determined using real-time RT-PCR with a slight modification (1). The virus titers ranged from 4.0 × 10^5^ to 11.4 × 10^11^ virus copies/g of stool. The detection range and sensitivity were equivalent to those of several other IC methods (6, 11, 12).

All of the isolated norovirus sequences belonged to GII.4 (Fig. 1C). The outbreak amino acid sequences were similar and closely matched the epidemic Sydney-2012 strain, having ~98% amino acid sequence identity. Three sporadic specimens (39-15, 99-15, and 101-15) were also similar (~98%) to the outbreak sequences. Two isolated sequences from sporadic patients (82-15 and 49-15) shared 90% amino acid sequence identity with the Sydney-2012 norovirus, and a BLAST search indicated that these sequences were related (~90%) to another GII.4 strain (ACL31340, Hunter virus) that circulated worldwide in 2004. These results indicated that the two novel capsid sequences represented a new genetic variant of GII.4.

**FIG 2:**
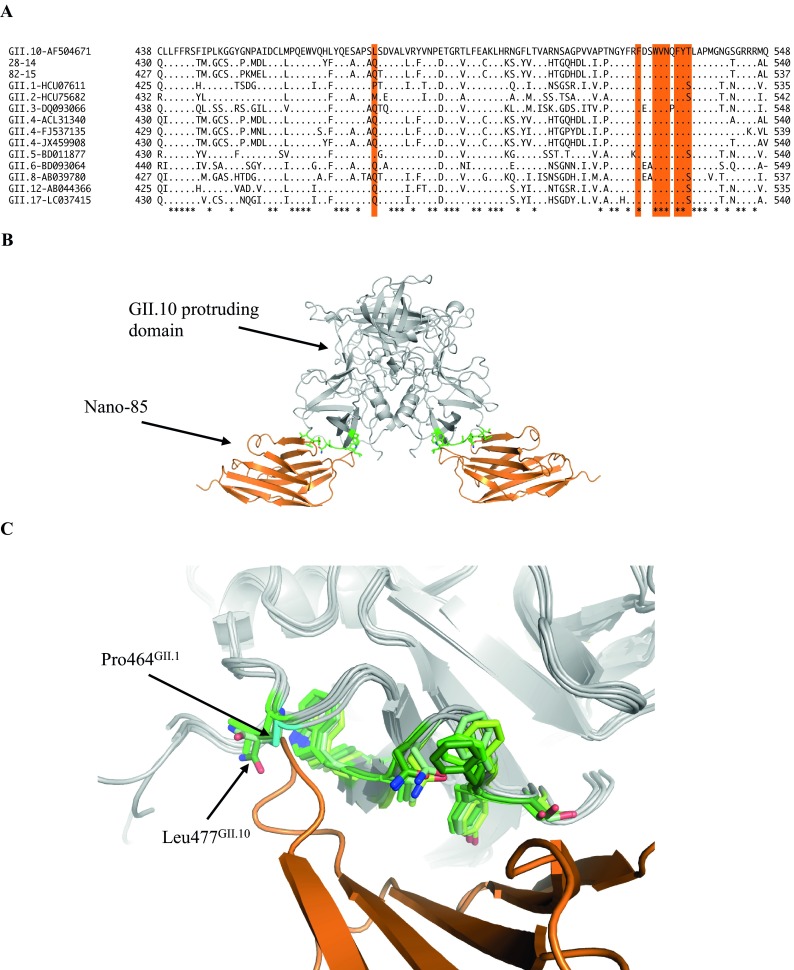
Sequence and structural analysis of the Nano-85 binding site. (A) Amino acid sequence alignment (partial shown) of two representative clinical specimens and other GII genotypes. The binding site of the Nano-85 is indicated (shaded orange) (9). (B) The GII.10 protruding domain (gray) and Nano-85 (orange) (4X7E). The GII.10 protruding domain residues interacting with Nano-85 are shown in green. (C) GII.1 (4ROX), GII.12 (3R6K), and GII.17 (5F4O) protruding domains (gray) were superpositioned on the GII.10 P domain Nano-85 complex. The protruding domain residues that likely interacted with Nano-85 are shown (GII.1, lemon; GII.10, green; GII.12, pale green; and GII.17, forest), and Pro464^GII.1^ was colored in cyan.

We also examined the GII genotype coverage by the Nano-IC method using VLPs from GII.1, GII.4 (two different clusters), GII.10, GII.12, and GII.17. All VLPs were prepared and purified as previously described (9). The VLPs were diluted to 100 µg/ml in the Nano-IC sample buffer. The Nano-IC assay showed positive readings for GII.4, GII.10, GII.12, and GII.17 (Fig. 1C). We previously showed that Nano-85 interacted with GII.10 residues Trp528, Asn530, Thr534, Leu477, Phe525, Val529, and Phe532 (9). A sequence alignment of other genotypes, as well as two representative clinical specimens (28-14 and 82-15) showed that the amino acids that interacted with Nano-85 were mostly conserved (Fig. 2A and B). The nonreactive GII.1 VLPs had identical amino acids, except at position Leu477^GII.10^, which was proline in GII.1 (Pro464). Based on the X-ray crystal structures of GII.1 P domain (PDB ID no. 4ROX) superpositioned on GII.10-Nano-85 complex structure (7X7D), the Pro464^GII.1^ appeared to clash with the main chain of Nano-85, indicating a steric hindrance in the binding (Fig. 2B and C).

An advantage of the Nano-IC method over conventional antibody-based IC methods is that atomic resolution structure of the Nanobody binding pockets can be easily obtained using X-ray crystallography, whereas complex structures of antibody binding pockets are often more difficult to determine (9, 13). This structural information can explain why Nanobodies bind to some strains and not others (see Fig. 2C). In addition, the Nanobodies can be readily produced in large quantities and are usually more stable than conventional antibodies (14). Overall, our results showed that the Nano-IC method could be useful in an outbreak setting. Detection methods that can rapidly detect index cases may help reduce the transmission of norovirus, especially in closed settings such as hospitals and cruise ships.

### Accession number(s).

The nucleotide sequences determined in this study have been deposited in GenBank under accession no. KU985152 to KU985166.
